# Supplementary material for: Mutation of Pseudomonas aeruginosa lasI/rhlI diminishes its cytotoxicity, oxidative stress, inflammation, and apoptosis on THP-1 macrophages
Source: Microbiol Spectr. 2024 Aug 20;12(10):e04146-23. doi: 10.1128/spectrum.04146-23 (PMC11448257; doi:10.1128/spectrum.04146-23)
Supplement: Supplementary material — Fig. S1 to S6; Videos S1 to S5; Tables S1 to S8. [file spectrum.04146-23-s0001.docx]

**Supplementary Materials**


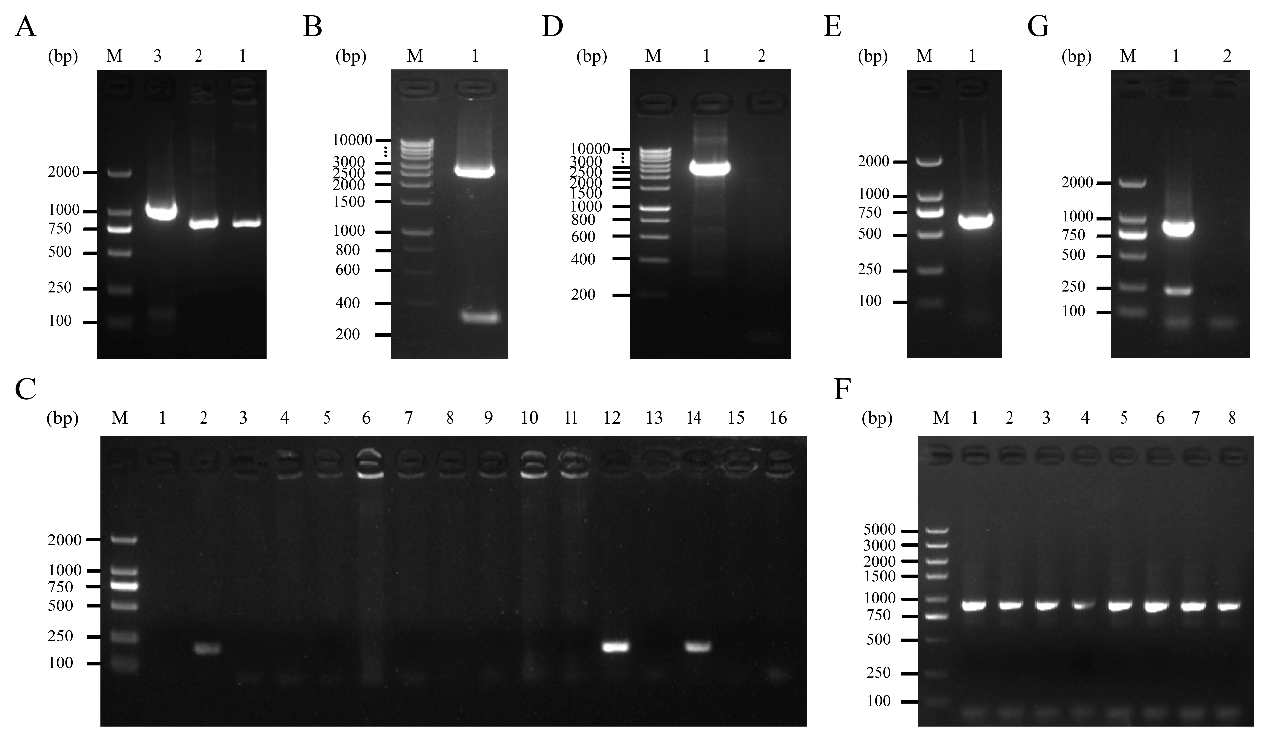


**FIG S1** Construction of *ΔlasI* and *ΔlasI-Comp.* of PAO1. (A) The PCR fragments of *lasI* homologous arms of PAO1 and Gm resistance gene. M: DNA marker; lane 1: upstream homologous arm, 820 bp; lane 2: downstream homologous arm, 853 bp; lane 3: Gm resistance gene fragment, 1078 bp. (B) Fusion PCR. M: DNA marker; lane 1: fusion fragment, 2691 bp. (C) Identification of internal primers. M: DNA marker; lane 1: no template negative control; lane 2: original strain; lane 3-11, 13 and 15-16: negative clones; lane 12 and 14: positive clones, 205 bp. (D) Identification of external primers. M: DNA marker; lane 1: No.1 negative clone, 2883 bp; lane 2: negative control. (E) The complementing fragment of *lasI*. M: DNA marker; lane 1: *lasI*, 660 bp. (F) Identification of the complementing plasmid pRK415-*lasI*. M: DNA marker; lane 1-8: positive clones, 920 bp. (G) Identification of complemented strain *ΔlasI-Comp.*. M: DNA marker; lane 1: positive clone; 2: *ΔlasI*, 920 bp.


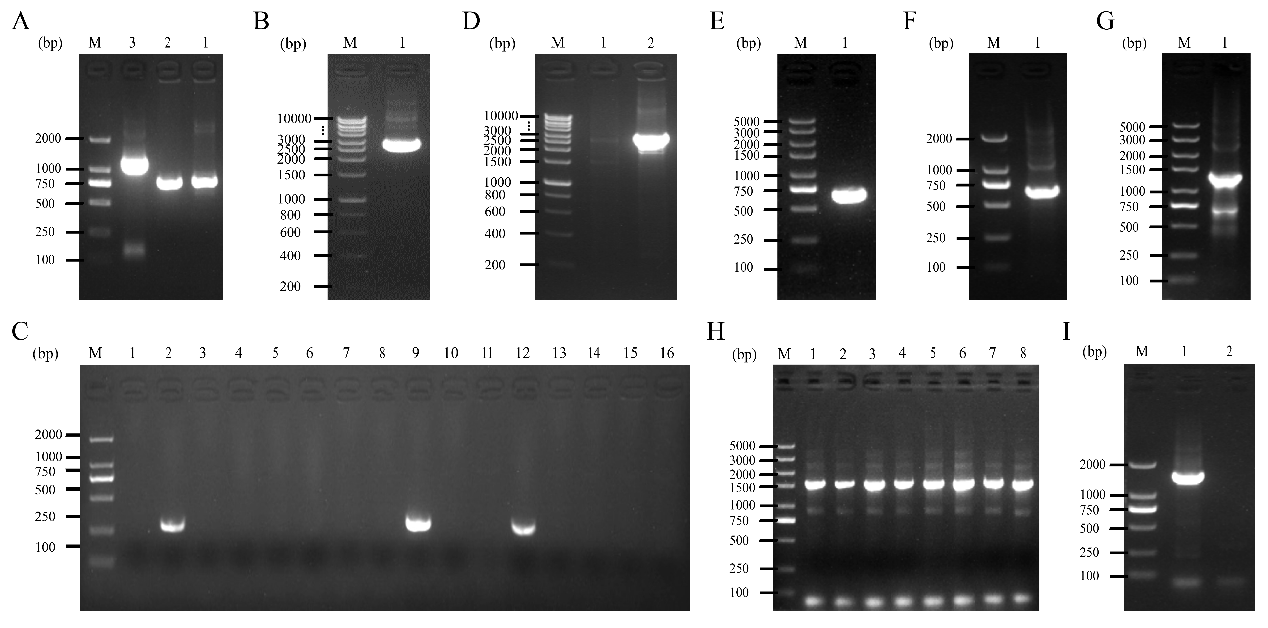


**FIG S2** Construction of *ΔlasIΔrhlI* and *ΔlasIΔrhlI-Comp.* of PAO1. (A) The PCR fragments of *rhlI* homologous arms of PAO1 and Apr resistance gene. M: DNA marker. lane 1: upstream homologous arm, 820 bp; lane 2: downstream homologous arm, 800 bp; lane 3: Apr resistance gene, 1218 bp. (B) Fusion PCR. M: DNA marker. lane 1: fusion fragment, 2787 bp. (C) Identification of internal primers. M: DNA marker. lane 1: no template negative control; lane 2: original strain; lane 3-8, 10-11 and 13-16: negative clones; lane 9 and 12: positive clones, 321 bp. (D) Identification of external primers. M: DNA marker. lane 1: negative control; lane 2: No. 1 negative clone, 2922 bp. (E) The complementing fragment of *lasI*. M: DNA marker; lane 1: *lasI*, 684 bp. (F) The complementing fragment of *rhlI*. M: DNA marker; lane 1: *rhlI*, 672 bp. (G) Fusion fragment of *lasI* and *rhlI*. M: DNA marker; lane 1: *lasIrhlI*, 1330 bp. (H) Identification of the complementing plasmid pRK415-*lasIrhlI*. M: DNA marker; lane 1-8: positive clones, 1576 bp. (I) Identification of complemented strain *ΔlasIΔrhlI -Comp.*. M: DNA marker; lane 1: positive clone; 2: *ΔlasIΔrhlI*, 1576 bp.

**
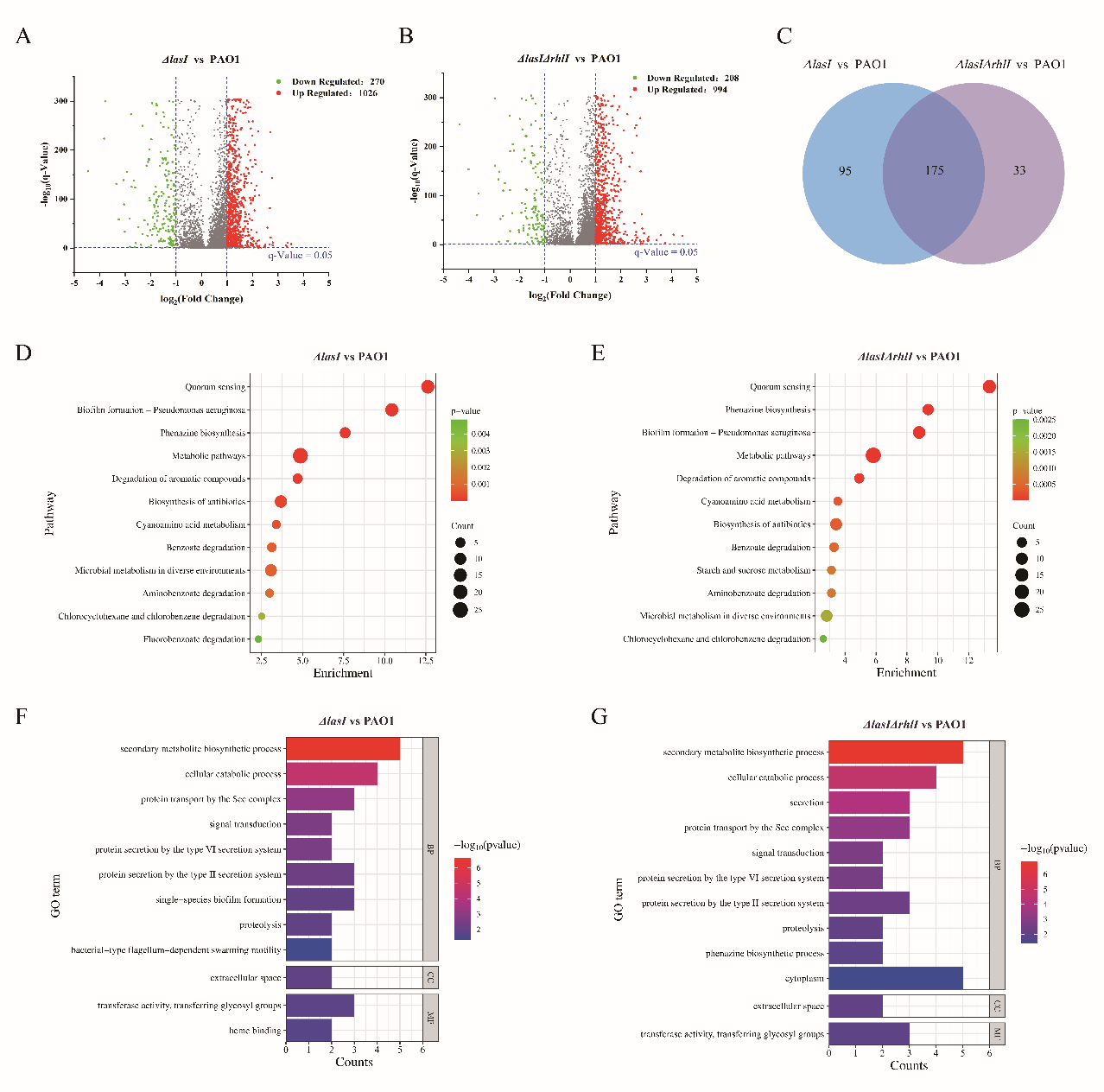
**

**FIG S3** DEGs, KEGG pathways and GO terms of different comparison groups based on RNA-seq. Volcano plot of DEGs in (A) *ΔlasI* versus PAO1 and (B) *ΔlasIΔrhlI* versus PAO1. The X-axis represents the fold change of gene expression, while the Y-axis represents the statistical significance of gene expression changes. A smaller q-Value corresponds to a larger -log(q-Value), indicating a more significant difference. Each point on the plot represents a gene, with downregulated genes depicted in green, upregulated genes shown in red, and non-differential genes displayed in gray. (C) Venn diagram of significantly downregulated genes in different comparison groups. The cumulative numbers within each large circle represent the total counts of downregulated DEGs in the comparison group. Overlapping regions indicate the shared downregulated DEGs, while non-overlapping regions represent uniquely downregulated DEGs. Bubble plot presenting the top 12 KEGG enrichment pathways for significantly downregulated genes in (D) *ΔlasI* versus PAO1 and (E) *ΔlasIΔrhlI* versus PAO1. The X-axis denotes the gene expression changes (-log(q-Value)) enriched to KEGG pathways, while the Y-axis indicates KEGG pathways. Enrichment bars display the top 12 GO terms for significantly downregulated genes in (F) *ΔlasI* versus PAO1 and (G) *ΔlasIΔrhlI* versus PAO1. The X-axis signifies the gene counts annotated to GO terms, whereas the Y-axis corresponds to GO terms. BP, biological process; CC, cellular component; MF, molecular function.

**
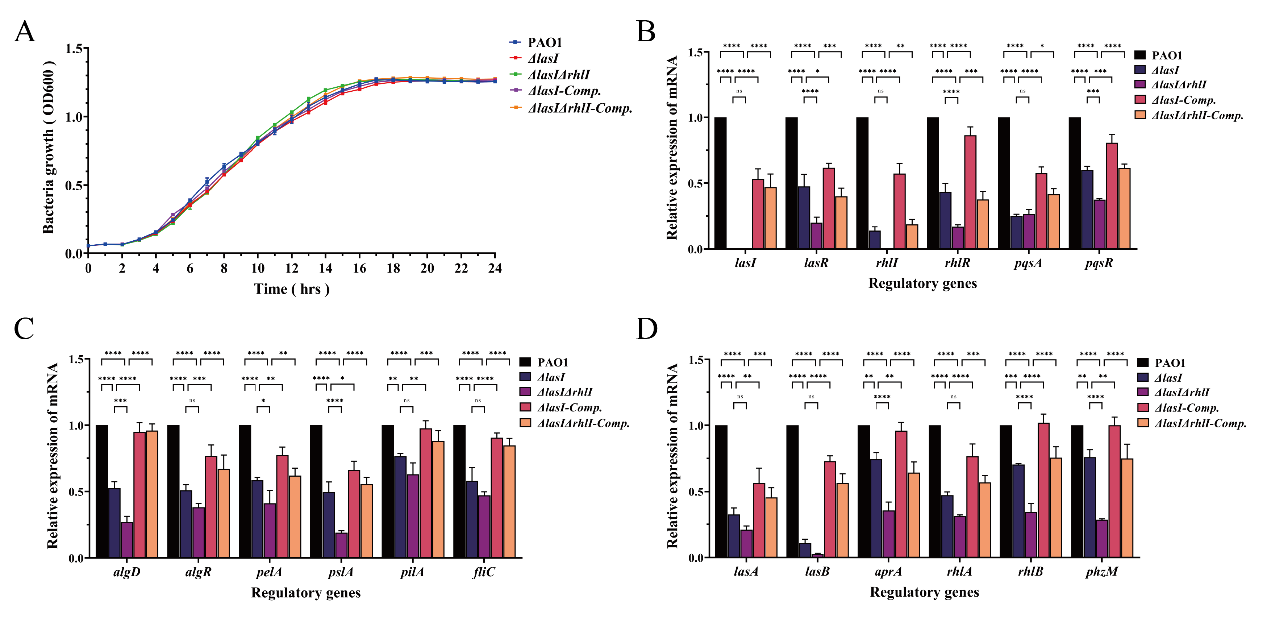
**

**FIG S4** Relative expression levels of key regulatory genes and related virulence genes of QS systems in PAO1, *ΔlasI*, *ΔlasIΔrhlI*, *ΔlasI-Comp.* and *ΔlasIΔrhlI-Comp.*. (A) 24 h time-growth curves. (B) Relative expression levels of *lasI*, *lasR*, *rhlI*, *rhlR*, *pqsA* and *pqsR*, key regulatory genes of the QS system. (C) Relative expression levels of the QS system related virulence genes *algD*, *algR*, *pslA*, *pelA*, *pilA* and *filC*. (D) Relative expression levels of the QS system related virulence genes *lasA*, *lasB*, *aprA*, *rhlA*, *rhlB* and *phzM*. All the data are presented as the M±SD of three independent experiments. *, *P<*0.05; **, *P<*0.01; ***, *P<*0.001; ****, *P<*0.0001; ns, no significance.

**
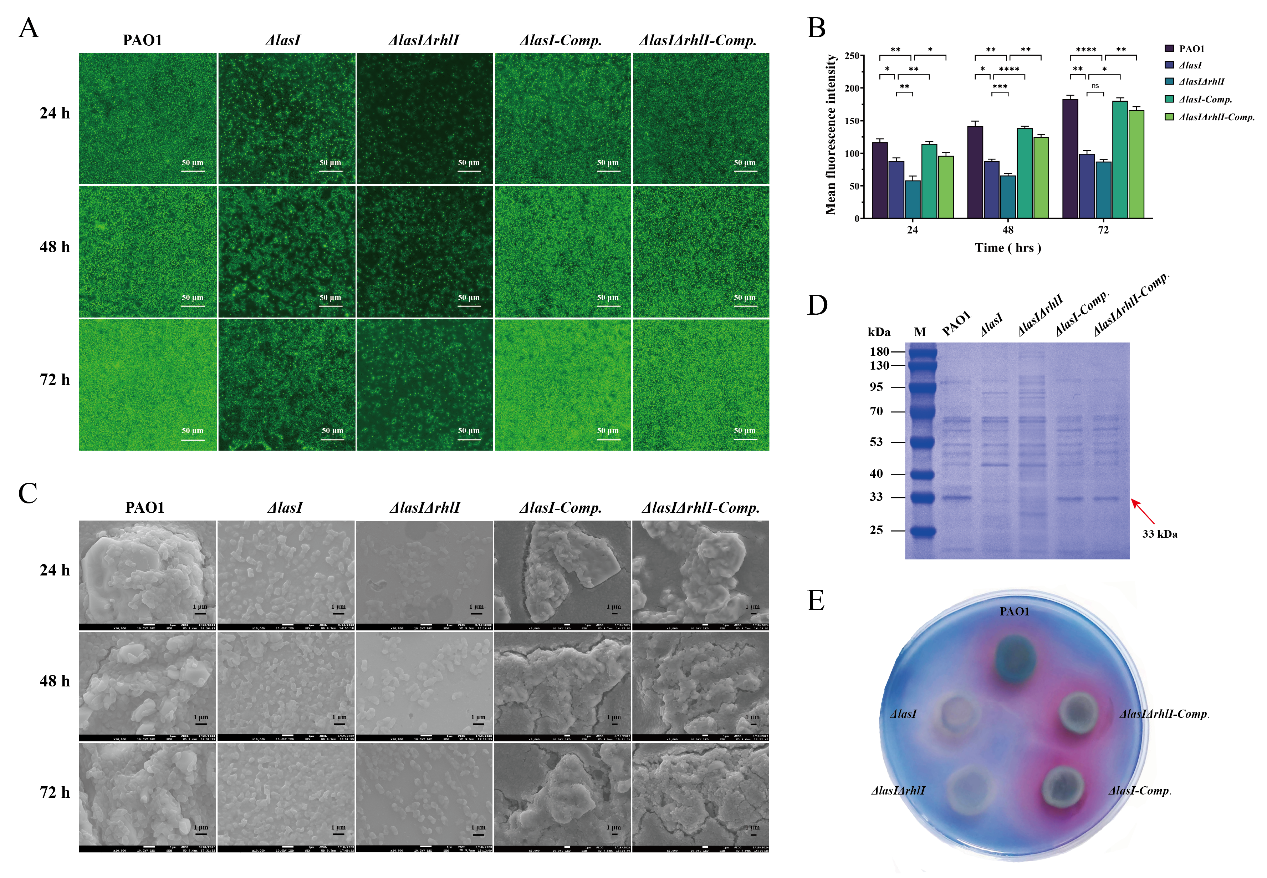
**

**FIG S5** The biofilm formation and EPS of PAO1, *ΔlasI*, *ΔlasIΔrhlI*, *ΔlasI-Comp.* and *ΔlasIΔrhlI-Comp.*. (A) Biofilm morphology observed by inverted fluorescence microscope and (B) the mean fluorescence intensity. Scale bar, 50 µm. (C) Biofilm morphology observed by SEM. Scale bar, 1 µm. (D) SDS-PAGE electrophoresis. M: protein marker. (E) Exopolysaccharide observed on Coomassie Brilliant Blue-Congo Red plate. All the data are presented as the M±SD of three independent experiments. *, *P<*0.05; **, *P<*0.01; ***, *P<*0.001; ****, *P<*0.0001; ns, no significance.

**
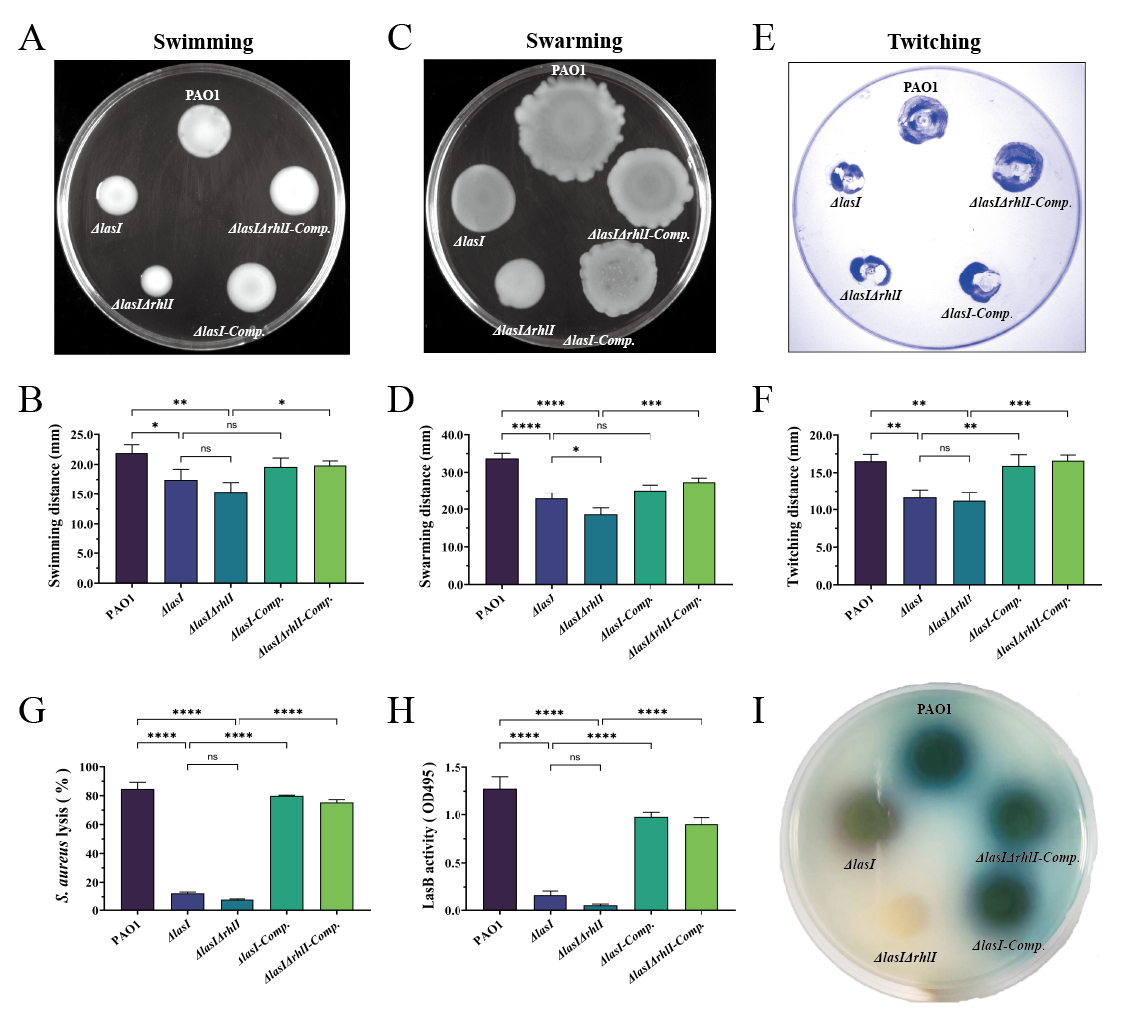
**

**FIG S6** The motility ability and virulence factors of PAO1, *ΔlasI*, *ΔlasIΔrhlI*, *ΔlasI-Comp.* and *ΔlasIΔrhlI-Comp.*. (A) Swimming plate assay and (B) swimming distance. (C) Swarming plate assay and (D) swarming distance. (E) Twitching plate assay and (F) twitching distance. (G) Lysis percentage of *S. aureus* ATCC 29213 at 150 min. (H) Elastin Congo red assay. (I) Pigment performance on PDP agar plate on day 5. All the data are presented as the M±SD of three independent experiments. *, *P<*0.05; **, *P<*0.01; ***, *P<*0.001; ****, *P<*0.0001; ns, no significance.

**Video S1** Tracking the 2D trajectory of PAO1 in LB liquid medium. Scale bar, 15 μm.

**Video S2** Tracking the 2D trajectory of *ΔlasI* in LB liquid medium. Scale bar, 15 μm.

**Video S3** Tracking the 2D trajectory of *ΔlasIΔrhlI* in LB liquid medium. Scale bar, 15 μm.

**Video S4** Tracking the 2D trajectory of *ΔlasI-Comp.* in LB liquid medium. Scale bar, 15 μm.

**Video S5** Tracking the 2D trajectory of *ΔlasIΔrhlI-Comp.* in LB liquid medium. Scale bar, 15 μm.

| **Table S1** Strains and plasmids used in this study | | |
| --- | --- | --- |
| **Materials** | **Genotype and relevant characteristics** | **Source** |
| **Strains** | | |
| *E. coli* DH5α λpir | F-φ80 lac ZΔM15Δ (lacZYA-arg F) LAMpir U169 endA1 recA1 hsdR17 (rk-, mk+) supE44λ -THI -1 gyrA96 relA1 phoA | This Lab |
| *E. coli* β2155 | strA hsdSΔ (lacZ) M15ΔdapA::erm pir::RP4 (::kan from SM10) | This Lab |
| *E. coli* TOP10 | **F- mcrA Δ(mrr-hsdRMS-mcrBC) φ80 lacZΔM15Δ lacX74recA1 araΔ139Δ(ara-leu)7697 galU galK rpsL (StrR)endA1nupG** | This Lab |
| *P. aeruginosa* PAO1 | ATCC15692 | This Lab |
| *ΔlasI* | PAO1, knockout of *lasI* gene | This study |
| *ΔlasIΔrhlI* | PAO1, knockout of *lasI* and *rhlI* gene | This study |
| *ΔlasI-Comp.* | *ΔlasI*, complementation of *lasI* gene | This study |
| *ΔlasIΔrhlI-Comp.* | *ΔlasIΔrhlI*, complementation of *lasI* and *rhlI* gene | This study |
| *S. aureus* | ATCC 29213 | This Lab |
| **Plasmids** | | |
| pUC57-Gm | Gentamicin ^+^ | This Lab |
| pUC57-Apr | Apramycin ^+^ | This Lab |
| pRK415-TC | Tetracycline ^+^ | This Lab |
| pCVD442 | Ampicillin ^+^ | This Lab |
| PCVD442-*ΔlasI*::Gm | pCVD442 derivative containing homologous arms of *lasI* gene of PAO1 | This study |
| PCVD442-*ΔrhlI*::Apr | pCVD442 derivative containing homologous arms of *rhlI* gene of PAO1 | This study |
| pRK415-*lasI* | pRK415 derivative containing complementing fragment of *lasI* gene of PAO1 | This study |
| pRK415-*lasIrhlI* | pRK415 derivative containing complementing fragment of *lasI* and *rhlI* gene of PAO1 | This study |

| **Table S2** Primer sequences used for constructing *ΔlasI* and *ΔlasIΔrhlI* mutants | | | |
| --- | --- | --- | --- |
| **Primers** | **Direction** | **Sequences (5′→3′)** | **Product sizes (bp)** |
| *lasI*-5’ | Forward | CATCTACCAGACGCGAAAGCAGCAC | 820 |
|  | Reverse | TACGATCATCTTCACTTCCTCCAAATAGGAAGCTG |  |
| *lasI*-3’ | Forward | GTTTCATGACGGGGACCTGTCGGC | 853 |
|  | Reverse | CTCAACGATAGCCAGGACTGGCACG |  |
| *lasI*-Gm | Forward | CAGCTTCCTATTTGGAGGAAGTGAAGATGATCGTAcatatgaatatcctccttagttcctattc | 1078 |
|  | Reverse | GCCGACAGGTCCCCGTCATGAAACgagctgcttcgaagttccta |  |
| *lasI*-in | Forward | CGATGGTTATGACGCACTCAGTC | 205 |
|  | Reverse | CTGTCCAGAGTTGATGGCGAAAC |  |
| *lasI*-out | Forward | GACGGTCAGTCACTGTACCCAGAGCGTAC | 2883 |
|  | Reverse | GTCGCCTATCTCGGTATCAGCGCGTTC |  |
| *rhlI*-5’ | Forward | GAAGTCGAAGGGTTGCTGCGGATGC | 820 |
|  | Reverse | GTGTGAGGTCGTCAGCCGTTTCGC |  |
| *rhlI*-3’ | Forward | TTCGATCATGACCAAGTCCCCGTGTCG | 800 |
|  | Reverse | GCTTCGATTACTACGCCTATGGCGTGC |  |
| *rhlI*-Apr | Forward | GCGAAACGGCTGACGACCTCACACgagctgcttcgaagttccta | 1218 |
|  | Reverse | CGACACGGGGACTTGGTCATGATCGAAcatatgaatatcctccttagttcctattc |  |
| *rhlI*-in | Forward | GAGGATCACGCCGTTGCGAACG | 321 |
|  | Reverse | GACCAGTTCGACCATCCGCAAACC |  |
| *rhlI*-out | Forward | CAGCCACTGGTCGACGTAGCGCTTG | 2922 |
|  | Reverse | GTAGCGAGATGCAGCCGATCCACGACAG |  |
| *lasI* | Forward | atatctagaATAAATTCTTCAGCTTCCTATTTGGAGGAAGTGAAGATG |  |
|  | Reverse 1 | atagaattcTCATGAAACCGCCAGTCGCTGTTC | 660 |
|  | Reverse 2 | ccgagaaaaaaaaggcggcatccctaTCATGAAACCGCCAGTCGCTGTTC | 684 |
| *rhlI* | Forward | tagggatgccgccttttttttctcgg | 672 |
|  | Reverse | atagaattctcacaccgccatcgacagcg |  |
| pRK415 | Forward | caacgcaattaatgtgagttagctcac | 299 |
|  | Reverse | ctcttcgctattacgccagctg |  |

| **Table S3** Regulatory genes and primer sequences of QS system of *P. aeruginosa* | | |
| --- | --- | --- |
| **Genes** | **Direction** | **Sequences (5′→3′)** |
| *lasI* | Forward | TGCGTGCTCAAGTGTTCAAGGAG |
|  | Reverse | GGACTGAGTGCGTCATAACCATCG |
| *lasR* | Forward | GCTGGAACGCTCAAGTGGAAAATTG |
|  | Reverse | TTCTCGTAGTCCTGGCTGTCCTTAG |
| *rhlI* | Forward | CTCTGAATCGCTGGAAGGGCTTTC |
|  | Reverse | TTTGCGGATGGTCGAACTGGTC |
| *rhlR* | Forward | GAGCGATACCAGATGCAGAACTACG |
|  | Reverse | TCCAGACCACCATTTCCGAGGAG |
| *pqsA* | Forward | GAAGTGAGCGAGGCGGTTCTG |
|  | Reverse | CTGTTCGGCGAGATGCTGGTC |
| *pqsR* | Forward | TCGTTCTGCGATACGGTGAG |
|  | Reverse | GCACTGGTTGAAGCGGGAG |
| *algD* | Forward | AGAAGTCCGAACGCCACA |
|  | Reverse | TCCAGCTCGCGGTAGAT |
| *algR* | Forward | AGACCGGCTACGGCTACA |
|  | Reverse | GCGTCGTGCTTCTTCAGTT |
| *pelA* | Forward | GGAACAGCCAGGTAATGGAC |
|  | Reverse | TCCAGGGTATCGAGGAACAG |
| *pslA* | Forward | GCCGCTTCATCCGCAAGAC |
|  | Reverse | ATTCGCTGACCGCCTCCTC |
| *pilA* | Forward | GATCAACCCGCTGAAGACC |
|  | Reverse | TGTTTCGGTCGCAGTAGAAG |
| *fliC* | Forward | CGACAAGGGTGTACTGACCA |
|  | Reverse | GACCTTCACTGCGACCTGAC |
| *lasA* | Forward | TGAATGACGACCTGTTCCTCTACG |
|  | Reverse | GCCGCTCCAGTGCTCCAG |
| *lasB* | Forward | CGAAGCCATCACCGAAGTCAAGG |
|  | Reverse | CAGCGATGTTGGCGACGAAATG |
| *aprA* | Forward | ACCGCTGCTGGACGACATC |
|  | Reverse | GTTGGAGTTGAAGCCGTACACC |
| *rhlA* | Forward | CTCGGTGGTGATGGCATTCG |
|  | Reverse | CGGTCTCGTTGAGCAGATGG |
| *rhlB* | Forward | TGTCACAACCGCACAGTATCG |
|  | Reverse | GACGCAGCCTTCAGCCATC |
| *phzM* | Forward | CACCGCACCACCGAGGAG |
|  | Reverse | CCTGGCAGCGACGATCATG |
| *16SrRNA* | Forward | CTCTTCCTGGTCGGCGAAAAGC |
|  | Reverse | GTTCGATACGCACCTGCCAGAG |

| **Table S4** Regulatory genes and primer sequences of human cytokines | | |
| --- | --- | --- |
| **Genes** | **Direction** | **Sequences (5′→3′)** |
| *IL-1α* | Forward | TGGTAGTAGCAACCAACGGGA |
|  | Reverse | ACTTTGATTGAGGGCGTCATTC |
| *IL-1β* | Forward | GCCAGTGAAATGATGGCTTATT |
|  | Reverse | AGGAGCACTTCATCTGTTTAGG |
| *IL-6* | Forward | ACTCACCTCTTCAGAACGAATTG |
|  | Reverse | CCATCTTTGGAAGGTTCAGGTTG |
| *IL-10* | Forward | GACTTTAAGGGTTACCTGGGTTG |
|  | Reverse | TCACATGCGCCTTGATGTCTG |
| *IL-12α* | Forward | CCTTGCACTTCTGAAGAGATTGA |
|  | Reverse | ACAGGGCCATCATAAAAGAGGT |
| *TNF-α* | Forward | CCTCTCTCTAATCAGCCCTCTG |
|  | Reverse | GAGGACCTGGGAGTAGATGAG |
| *COX-2* | Forward | GGTTGCTGGTGGTAGGAATGTTC |
|  | Reverse | CTGGTATTTCATCTGCCTGCTCTG |
| *iNOS* | Forward | ATCCAGCCGTGCCACCATC |
|  | Reverse | CCATCCACCACTCGCTCCAG |
| *SOD2* | Forward | CACCGAGGAGAAGTACCAGGAG |
|  | Reverse | CCACCACCGTTAGGGCTGAG |
| *GPX4* | Forward | CGCTGTGGAAGTGGATGAAGATC |
|  | Reverse | TGTCGATGAGGAACTGTGGAGAG |
| *IκBα* | Forward | CCCGCACCTCCACTCCATCC |
|  | Reverse | AGCATTGACATCAGCACCCAAGG |
| *NF-κB p65* | Forward | CCAGACCAACAACAACCCCTTCC |
|  | Reverse | AAGCAGAGCCGCACAGCATTC |
| *Bax* | Forward | GATTGCCGCCGTGGACACAG |
|  | Reverse | CCAGTTGAAGTTGCCGTCAGAAAAC |
| *Bcl-2* | Forward | TACGAGTGGGATGCGGGAGATG |
|  | Reverse | CCGGGCTGGGAGGAGAAGATG |
| *β-actin* | Forward | CATGTACGTTGCTATCCAGGC |
|  | Reverse | CTCCTTAATGTCACGCACGAT |

| **Table S5** Effects of *lasI*/*rhlI* gene deletion on virulence phenotype of *P. aeruginosa* | | | | | | | |
| --- | --- | --- | --- | --- | --- | --- | --- |
| **Items** | **PAO1** | ***ΔlasI*** | ***ΔlasIΔrhlI*** | ***ΔlasI-Comp.*** | ***ΔlasIΔrhlI-Comp.*** | ***F*** | ***P*** |
| Swimming distance (mm) | 21.90±1.39 | 17.43±1.78 | 15.27±1.47 | 19.6±1.473 | 19.83±0.76 | 8.897 | 0.0025 |
| Swarming distance (mm) | 33.70±1.38 | 22.90±1.51 | 18.63±1.72 | 25.10±1.51 | 27.37±1.11 | 43.83 | < 0.0001 |
| Twitching distance (mm) | 16.53±0.91 | 11.33±1.06 | 11.17±1.17 | 15.90±1.49 | 16.60±0.75 | 18.23 | < 0.0001 |
| Average speed (μm/s) | 11.17±1.16 | 8.33±0.70 | 6.73±0.68 | 10.50±0.60 | 9.37±0.62 | 15.26 | 0.0003 |
| Average displacement (μm) | 0.34±0.02 | 0.27±0.02 | 0.25±0.01 | 0.34±0.03 | 0.32±0.03 | 9.08 | 0.0023 |
| *S. aureus* lysis (%) | 84.62±4.65 | 12.43±0.86 | 7.38±0.51 | 79.90±0.45 | 75.28±2.00 | 832.3 | < 0.0001 |
| LasB activity (OD495nm) | 1.28±0.12 | 0.17±0.04 | 0.05±0.01 | 0.98±0.05 | 0.90±0.07 | 180.0 | < 0.0001 |

Note: all the data are presented as the M±SD of three independent experiments.

| **Table S6** Effects of *lasI*/*rhlI* gene deletion on relative protein expression levels in THP-1 macrophages | | | | | | | | |
| --- | --- | --- | --- | --- | --- | --- | --- | --- |
| **Protein** | **Control** | **PAO1** | ***ΔlasI*** | ***ΔlasIΔrhlI*** | ***ΔlasI-Comp.*** | ***ΔlasIΔrhlI-Comp.*** | ***F*** | ***P*** |
| SOD2 | 1.00±0.00 | 0.36±0.01 | 0.95±0.05 | 1.01±0.07 | 0.63±0.02 | 0.34±0.02 | 235.5 | < 0.0001 |
| GPX4 | 1.00±0.00 | 0.70±0.02 | 0.89±0.01 | 0.76±0.04 | 0.46±0.07 | 0.30±0.06 | 116.1 | < 0.0001 |
| COX-2 | 1.00±0.00 | 2.88±0.37 | 2.10±0.17 | 1.78±0.16 | 2.02±0.16 | 2.25±0.08 | 30.87 | < 0.0001 |
| iNOS | 1.00±0.00 | 7.47±0.63 | 3.75±0.20 | 1.90±0.07 | 3.91±0.22 | 5.23±0.18 | 187.5 | < 0.0001 |
| P-IκBα/ IκBα | 1.00±0.00 | 1.20±0.05 | 0.30±0.01 | 0.19±0.01 | 1.24±0.06 | 0.59±0.07 | 353.1 | < 0.0001 |
| P-p65/p65 | 1.00±0.00 | 1.67±0.06 | 0.68±0.01 | 0.60±0.01 | 1.01±0.02 | 0.70±0.05 | 424.7 | < 0.0001 |
| Bax | 1.00±0.00 | 1.34±0.03 | 1.18±0.02 | 1.05±0.01 | 1.32±0.05 | 1.33±0.02 | 93.77 | < 0.0001 |
| Bcl-2 | 1.00±0.00 | 0.41±0.02 | 0.80±0.03 | 0.95±0.03 | 0.17±0.02 | 0.34±0.01 | 883.4 | < 0.0001 |
| Bcl-2/ Bax | 1.00±0.00 | 0.31±0.02 | 0.72±0.04 | 0.76±0.04 | 0.14±0.01 | 0.26±0.01 | 536.3 | < 0.0001 |

Note: all the data are presented as the M±SD of three independent experiments.

| **Table S7** Effects of *lasI*/*rhlI* gene deletion on relative mRNA expression levels in THP-1 macrophages | | | | | | | | |
| --- | --- | --- | --- | --- | --- | --- | --- | --- |
| **Gene** | **Control** | **PAO1** | ***ΔlasI*** | ***ΔlasIΔrhlI*** | ***ΔlasI-Comp.*** | ***ΔlasIΔrhlI-Comp.*** | ***F*** | ***P*** |
| *SOD2* | 1.00±0.00 | 0.47±0.08 | 0.88±0.08 | 1.04±0.13 | 0.80±0.06 | 0.72±0.06 | 13.98 | 0.0030 |
| *GPX4* | 1.00±0.00 | 0.59±0.05 | 0.96±0.04 | 0.97±0.08 | 0.56±0.04 | 0.60±0.08 | 31.79 | 0.0003 |
| *IL-1α* | 1.00±0.00 | 41.53±2.24 | 26.45±0.52 | 18.62±1.91 | 37.71±2.77 | 30.14±2.95 | 101.9 | < 0.0001 |
| *IL-1β* | 1.00±0.00 | 10.53±1.39 | 6.82±0.13 | 4.61±0.56 | 9.18±0.90 | 7.01±0.17 | 44.55 | < 0.0001 |
| *IL-6* | 1.00±0.00 | 81.04±3.18 | 45.29±2.44 | 29.89±7.25 | 61.69±4.53 | 49.24±3.38 | 89.71 | < 0.0001 |
| *IL-10* | 1.00±0.00 | 3.85±0.21 | 4.13±0.12 | 4.25±0.27 | 2.91±0.17 | 3.22±0.02 | 109.2 | < 0.0001 |
| *IL-12* | 1.00±0.00 | 7.84±0.04 | 4.83±0.38 | 4.43±0.26 | 6.24±0.28 | 6.16±0.57 | 106.1 | < 0.0001 |
| *TNF-α* | 1.00±0.00 | 28.95±1.28 | 21.05±0.83 | 19.30±0.57 | 25.40±1.87 | 24.02±1.29 | 150.4 | < 0.0001 |
| *COX2* | 1.00±0.00 | 106.61±6.27 | 57.06±9.19 | 28.07±1.51 | 32.35±1.59 | 71.17±6.62 | 96.02 | < 0.0001 |
| *iNOS* | 1.00±0.00 | 10.00±0.59 | 5.80±0.77 | 3.73±0.58 | 5.70±0.17 | 7.78±0.95 | 53.12 | < 0.0001 |
| *IκBα* | 1.00±0.00 | 14.58±0.79 | 6.87±1.11 | 5.51±0.43 | 14.19±1.04 | 11.78±1.10 | 81.56 | < 0.0001 |
| *NF-κB p65* | 1.00±0.00 | 12.57±0.92 | 6.18±0.91 | 2.81±0.27 | 9.02±0.75 | 9.28±1.04 | 66.57 | < 0.0001 |
| *Bax* | 1.00±0.00 | 10.75±0.63 | 5.74±0.39 | 4.28±0.21 | 8.67±0.17 | 10.70±0.16 | 277.8 | < 0.0001 |
| *Bcl-2* | 1.00±0.00 | 0.51±0.04 | 1.12±0.07 | 1.34±0.02 | 0.68±0.02 | 0.78±0.05 | 113.0 | < 0.0001 |

Note: all the data are presented as the M±SD of three independent experiments.

| **Table S8** Effects of *lasI*/*rhlI* gene deletion on protein levels of inflammatory cytokines in THP-1 macrophages | | | | | | | | |
| --- | --- | --- | --- | --- | --- | --- | --- | --- |
| **Protein** | **Control** | **PAO1** | ***ΔlasI*** | ***ΔlasIΔrhlI*** | ***ΔlasI-Comp.*** | ***ΔlasIΔrhlI-Comp.*** | ***F*** | ***P*** |
| IL-1α (pg/ml) | 842.67±65.39 | 1922.83±64.92 | 1535.99±23.90 | 1259.49±84.80 | 1707.32±35.83 | 1568.16±61.53 | 120.4 | < 0.0001 |
| IL-1β (pg/ml) | 12.61±1.22 | 108.42±6.96 | 66.99±5.26 | 46.42±3.35 | 91.22±6.73 | 74.75±10.85 | 82.07 | < 0.0001 |
| IL-6 (pg/ml) | 81.04±9.16 | 130.30±9.29 | 97.28±6.83 | 87.28±6.01 | 112.98±7.15 | 105.67±3.69 | 18.26 | < 0.0001 |
| IL-10 (pg/ml) | 20.02±2.08 | 32.41±2.97 | 34.64±1.90 | 39.61±1.83 | 30.04±1.97 | 32.04±1.15 | 29.96 | < 0.0001 |
| IL-12 (pg/ml) | 79.72±8.63 | 504.38±28.55 | 283.55±13.96 | 220.80±18.90 | 403.86±29.45 | 337.03±29.15 | 124.0 | < 0.0001 |
| TNF-α (pg/ml) | 6.62±0.31 | 16.62±1.09 | 11.35±1.17 | 9.52±0.79 | 17.05±0.78 | 14.63±1.40 | 53.31 | < 0.0001 |

Note: all the data are presented as the M±SD of three independent experiments.
